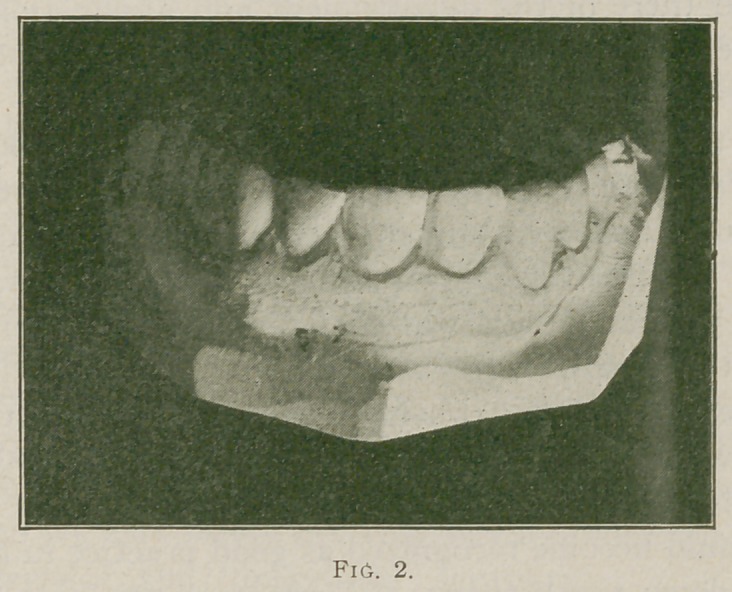# Porcelain Jacket Crowns

**Published:** 1907-09-15

**Authors:** John E. Roche


### PORCELAIN JACKET CROWNS.

BY JOHN E. ROCHE, D.D.S

Demonstrated at the Michigan Dental Association meeting, June 5, 1907, with a practical case.

The case treated was for a lady the enamel of whose teeth was badly pitted and defective, see cut No. 1 for condition before and cut No. 2 for after treatment.

The method: First, trim the tooth surfaces down leaving a shoulder slightly under the gum margin, as for Spalding jacket crown. Care should be taken not to trim the shoulder too deeply, as there is a liability to chip off what enamel there is left. Aslo leave the tooth as long as possible and slightly cone shaped. This will afford good protection for the pulp.

Fig. 1.

Next, take an impression in matrix platinum, then select a postless crown the proper shade, size, etc., and trim it out to fit over the matrix as near as possible. This can be done with a small coarse carborundum stone and plenty of water, one can be cut out in from fifteen to twenty minutes.

Next, mix some porcelain to a creamy consistency and fill the crown half full, and with your matrix in place and thoroughly dried, force it to place; this packs the porcelain, so that when baked there is but a slight amount of shrinkage. The matrix will then come away readily with the crown. Place in furnace and bake to a biscuit; then proceed with filling and baking in the regular way, it may require two or three more bakings to make the crown complete.

Advantages: A much stronger crown than one made from a facing; because the porcelain is not so well fused and the facing is liable to separate or be broken off; while in this case we have a crown that has been baked in one piece originally.

FIG. 2.

The cuts show the case as it was exhibited at the clinic, it consists of the six upper incisor and cuspid teeth from cuspid to cuspid, inclusive. They have been on about one year and a half, the pulps were not destroyed in any of the teeth, and the patient reports that they have not caused her the slightest discomfort. The gums have retained their normal color and healthy condition. I think this is largely due to the fact that the shoulder is but slightly under the gum margin and there are no overhanging edges. The gums should be no more likely to recede in this than in a normal case. If the baking is carefully done these crowns should be as strong as the Spalding jacket crown, and are considerably less trouble to make.

## Figures and Tables

**Fig. 1. f1:**
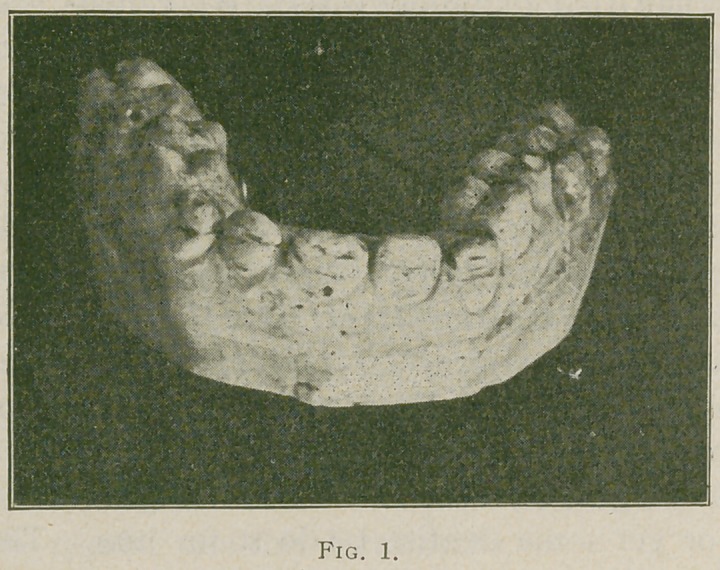


**Fig. 2. f2:**